# Wearing Cushioning Shoes Reduce Load Rates More Effectively in Post-Fatigue than in Pre-Fatigue during Landings

**DOI:** 10.3390/biology10100962

**Published:** 2021-09-26

**Authors:** Xi Wang, Liqin Deng, Wing-Kai Lam, Yang Yang, Xini Zhang, Weijie Fu

**Affiliations:** 1 School of Kinesiology, Shanghai University of Sport, Shanghai 200438, China; 1811516022@sus.edu.cn (X.W.); 1921516007@sus.edu.cn (L.D.); 1911516023@sus.edu.cn (Y.Y.); 1911516024@sus.edu.cn (X.Z.); 2 Shanghai Frontiers Science Research Base of Exercise and Metabolic Health, Shanghai University of Sport, Shanghai 200438, China; 3 Department of Kinesiology, Shenyang Sport University, Shenyang 110102, China; gilbertlam@li-ning.com.cn; 4 Li Ning Sports Science Research Center, Li Ning (China) Sports Goods Company, Beijing 101111, China; 5 Key Laboratory of Exercise and Health Sciences of Ministry of Education, Shanghai University of Sport, Shanghai 200438, China

**Keywords:** fatigue, sports shoe, impact, kinematics, drop landing

## Abstract

**Simple Summary:**

Athlete experience high impact forces during landing, which is a contributing factor to injury risk potentials. As a potential factor of affecting the impact force, previous study of the effects of footwear cushioning effect on landing biomechanics were inconsistent. Furthermore, limited efforts have been exerted on the relationship between footwear cushioning and fatigue. In this study, the footwear cushioning effects on bipedal landing biomechanics before and after acute exercise-induced fatigue protocol were explored. The results of this study suggest that footwear cushioning can reduce landing-related rearfoot impact forces regardless of fatigue conditions. In a situation where the neuromuscular activity is reduced or absent, e.g., post-fatigue, wearing better cushioning shoes show superior attenuation, as indicated by low forefoot and rearfoot impacts.

**Abstract:**

Purpose: this study aimed to investigate the footwear cushioning effects on impact forces and joint kinematics of the lower extremity during bipedal drop landings before and after acute exercise-induced fatigue protocol. Methods: in this case, 15 male collegiate basketball athletes performed drop landings from a 60 cm platform wearing highly-cushioned shoes (HS) and less cushioned shoes (control shoes, CS) before and after acute fatigue-inducing exercises (i.e., shuttle run combined with multiple vertical jumps). Force plates and motion capturing systems were synchronised to measure ground reaction forces and kinematic data during drop landings. Maximum jump height was analysed with one-way ANOVA. Two-way repeated measure ANOVAs were performed on each of the tested variables to examine if there was significant main effects of shoe and fatigue as well as the interaction. The significance level was set to 0.05. Results: rearfoot peak impact forces and loading rates significantly reduced when the participants wore HS in pre- and post-fatigue conditions. The peak loading rates in forefoot significantly reduced when HS were worn in post-fatigue. Compared with pre-fatigue, wearing HS contributed to with 24% and 13% reduction in forefoot and rearfoot peak loading rates, respectively, and the occurrence times of first and second peak impact forces and loading rates were much later. In the post-fatigue, a significant increase in the initial contact and minimum angles of the ankle were observed in HS compared with CS. Conclusion: these findings suggest that footwear cushioning can reduce landing-related rearfoot impact forces regardless of fatigue conditions. In a situation where the neuromuscular activity is reduced or absent such as post-fatigue wearing better cushioning shoes show superior attenuation, as indicated by lower forefoot and rearfoot impacts.

## 1. Introduction

Athletes may experience impact forces up to 3.5 times to 6 times of his/her body weight (BW) during various landings in basketball and volleyball [1,2]. Previous studies have shown that excessive impact on the lower extremity is one of the contributing factors to injury risk potentials [3,4,5].

Factors, such as footwear characteristics, are known to affect impact loading [6]. There are many different types of footwear available in the market nowadays [7], e.g., minimalist and highly cushioned footwear, claiming to reduce or prevent injury [8]. From the perspective of mechanics and materials science, sports footwear should be capable of reducing impact forces exerted on the lower extremity [1]. Previous studies have reported that wearing different shoelace patterns shows different peak loading rates [9]; meanwhile the use of shoes with an incorrect length can cause foot injuries [10]. Moreover, it was reported that wearing cushioned shoes could reduce ground reaction forces (GRFs) [11] and vertical loading rates (LRs) [12], which have been hypothesised to reduce running-related injury risks, such as stress fractures [13]. For the individuals with foot and motor system dysfunction, cushioned footwear was essential. Previous studies have been reported the reduction in plantar pressure with the use of cushioning materials plays an important role in the clinical management of the diabetic foot [14]. Meanwhile, the cushioned footwear worn by individuals with ankle arthropathy has a significant effect on the amount of force acting at the joint [15]. However, understanding the shoe construction and materials used cannot completely explain the cushioning performance efficacy of sport shoes because the neuromuscular system can play a role in reducing the impacts by changing its movement characteristics [16] and joint compliance [17] in response to various shoe conditions. These studies have shown no causal relationship found between impact forces and running injuries regardless of midsole hardness [16] and insert designs [18]. To date, whether footwear cushioning has detrimental or beneficial effects could be related to test parameters, movement tasks and physical conditions of participants across various studies [1,19,20]. Therefore, few scientific guidelines have been established to understand the footwear cushioning effects on impact attenuation and injury prevention.

Our previous study [6] found that when the participants were aware and neuromuscular system was ready for self-initiated drop landing, wearing highly-cushioned shoes (HS) did not show cushioning performance benefits (i.e., reduced peak impact force and LR) compared with wearing low cushioning shoes. Interestingly, participants wearing better cushioning shoes experience lower impact force and LR than that of wearing less cushioning shoes when performing an unanticipated landing [6]. This finding suggested that footwear cushioning effect could become significant and discriminative when the ability of neuromuscular control becomes weaker. The performance benefits of sports equipment could be related to the awareness of and confidence in footwear modifications [21], especially when participants have poor postural balance after acute exercise-induced fatigue protocol [22].

After long-term and high-intensity exercises can inevitably induce fatigue, which is associated with muscle damage (creatine kinase and myoglobin) and inflammation (C-reactive protein, leukocytes and cytokines) markers during a basketball match [23]. Fatigue can reduce the excitability of the central nervous system, which is related to musculoskeletal response delay [24] and change in joint mechanics and motor control [25]. These changes may increase the incidence of sport injuries [24]. To date, most fatigue and footwear studies have predominantly focused on running economy [26], subjective comfort [27] and balance control [28]. However, limited efforts have been exerted on the relationship between footwear cushioning and fatigue in basketball. The footwear cushioning effect on landing biomechanics associated with physical fatigue should be studied because shoes are considered an important interface between the foot and ground.

This study aimed to investigate the shoe effects (Highly-cushioned shoe, HS vs. control shoes, CS) on impact forces and joint kinematics during drop landing before and after acute exercise-induced fatigue protocol. HS would reduce the vertical GRFs and LRs in post-fatigue but not in pre-fatigue condition. Participants wearing HS would demonstrate large changes in joint angle and range of motion (RoM) between pre- and post-fatigue conditions compared with wearing CS.

## 2. Materials and Methods

### 2.1. Participants

In this case, 15 male collegiate basketball athletes with a national second-class level (age: 22.1 ± 1.7 years; height: 179.3 ± 3.2 cm; body mass: 72.2 ± 5.5 kg) were recruited randomly in this study by distributing leaflets on campus and through the online platform. A priori power analysis was performed to indicate the statistical power revealing a sample size of 15 was sufficient to minimize the probability of Type II error [29,30]. All participants had an average training of 5.7 years for regular competition. The exclusion criteria: participants (1) have experienced any lower extremity injuries in the past six months; (2) had or were having the neuromuscular disease; (3) engaged in strenuous training within 24 h. All the participants signed informed consent, and ethical approval was granted by the Institutional Review Board of the university prior to the study (No. 2017007).

### 2.2. Experimental Shoes

**Figure 1:**
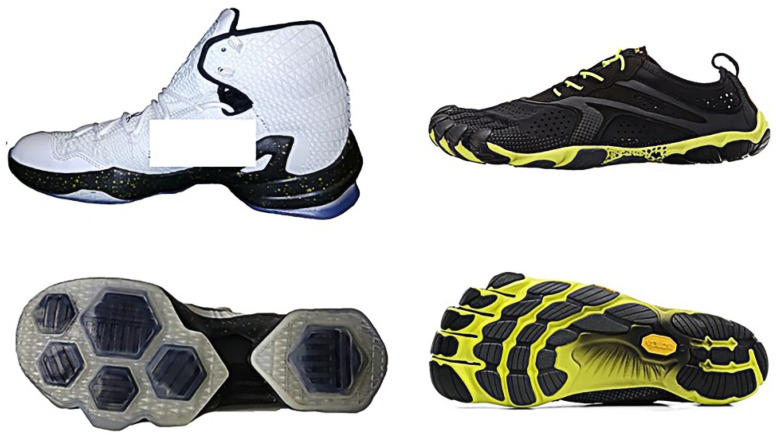
Highly-cushioned shoes, HS (**left**) and less cushioned shoes, CS (**right**).

Highly-cushioned shoe (HS) and control shoes (CS) were selected because of their extreme difference in cushioning properties (Figure 1). In particular, HS (Lebron James 13 Elite, Nike, Beaverton, OR, USA) were marketed with superior cushioning performance for basketball players. HS had a durable Kurim cage upper, carbon fiber shank, full-length bootie and six hexagonal Zoom Air-sole units on each shoe. The midsole thickness is 12–35 mm and the hardness of the midsole and outsole of the basketball shoes was 66 shore C and 50 shore A, respectively. CS (Vibram Five Fingers Bikila EVO, Vibram, Albizzate, Italy) were marketed with minimal cushioning performance in the footwear industry. CS had no shoe upper support, with 3 mm total sole thickness and zero heel-to-toe drop. The hardness of outsole of the control shoes was 60 shore A. In this study, CS were made to mimic barefoot condition without leaving the foot completely unprotected during the landing task. US sizes of 8.5 and 9.0 were used to increase the number of participants recruited in this study.

### 2.3. Experimental Procedure

**Figure 2:**
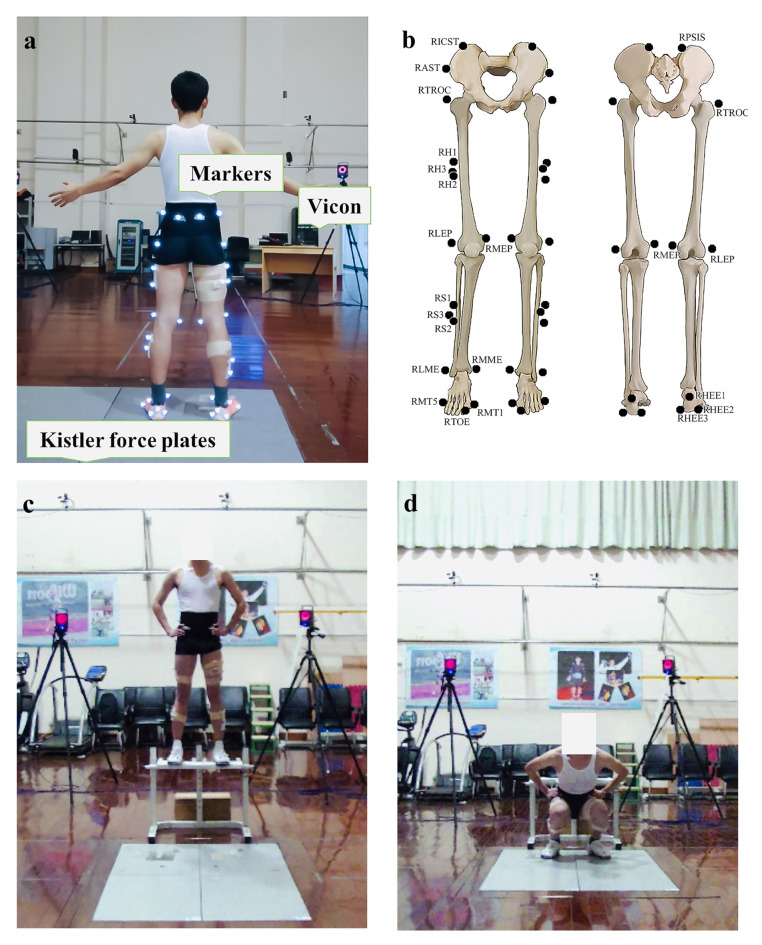
(**a**) experimental setup; (**b**) marker placements; (**c**,**d**) landing manoeuvre.

**Figure 3:**
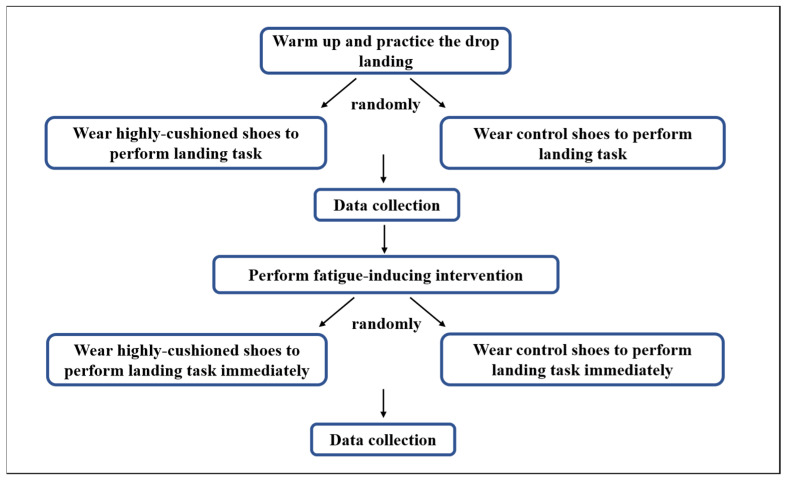
Diagram of the experimental flow chart.

All participants performed the first landing evaluation session, fatigue-inducing protocol and second landing evaluation session. On arrival, each participant performed jogging and stretching for 5–10 min and was given sufficient practice time to familiarise with the drop landing. In this case, 40 reflective markers were attached on the lower extremities to define hip, knee, ankle joints and the biomechanical model of the participants (Figure 2). Subsequently, participants were instructed to stand upright and look forward whilst positioning their arms on their hips to reduce postural sway. Landing task was initiated by dropping with both legs from a 60 cm platform and then landing with each leg on the separated force plates [30,31] (Figure 2). A successful trial was regarded as a trial with a toe-to-heel landing pattern on two force platform (9287B, Kistler Corporation, Switzerland) (vertical stiffness: 30 N/um) with clean footfalls and good balance [32]. The orders of shoes were randomised across participants. Then, the fatigue-inducing intervention was executed, that is, shuttle runs combined with multiple vertical jumps. The rating of perceived exertion (RPE) scale was immediately completed. After completion of fatigue-inducing intervention, the second landing section was immediately conducted for two shoe conditions in a randomised order (Figure 3).

### 2.4. Acute Exercise-Induced Fatigue Protocol

**Figure 4:**
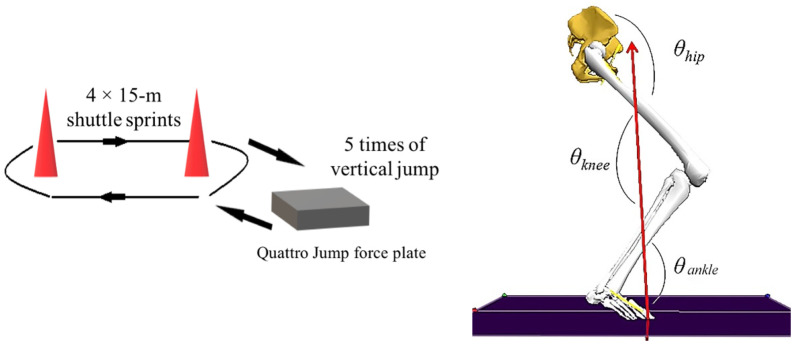
Scheme of fatigue-inducing protocol and joint angle definitions.

The maximum vertical jump height of each participant was measured before conducting the fatigue-inducing exercise intervention. The protocol comprised five consecutive vertical jumps, followed by a set of 4 × 15-m shuttle sprints [30] (Figure 4). Participants were required to repeat the aforementioned sequence with their maximum effort until the intervention was completed. The inclusion criteria of reaching a fatigued state included: (1) participants’ jumping heights were less than the 70% maximum height for all five jumps [30], and (2) their heart rate (HR) reached 90% of their maximum HR using the formula (Maximum HR = 220−age) [33].

During the fatigue-inducing protocol, HR was monitored with an HR transmitter belt monitor (SS020674000, Suunto Oy, Vantaa, Finland) attached to the participants’ chest during the entire procedure. RPE was collected to immediately evaluate the stage of exertion after fatigue-inducing exercises [32]. The maximum vertical jump height after fatigue intervention was measured using a Quattro Jump force plate (9290BD, Kistler Corporation, Winterthur, Switzerland).

### 2.5. Data Processing

The sagittal kinematic and GRF data of the landing leg (defined as the landing leg in single lay-up jump manoeuvre) were collected using synchronised 10-infrared camera motion capture system (Vicon T40, Oxford Metrics, Oxford, UK, sampling at 240 Hz) and 90 cm × 60 cm force plates (9287B, Kistler Corporation, Winterthur, Switzerland, sampling at 1200 Hz). The trajectory data were filtered through a Butterworth fourth-order, low-pass filter at 7 Hz cut-off frequency via Visual 3D software (v. 4.96.13, C-Motion Inc., Germantown, MD, USA) [34].

The impact force variables include the first peak (Fz_1max_) and second peak (Fz_2max_) vertical GRF (vGRF) during the time interval from initial foot contact to the minimum angle occurrence of the knee joint, the time to Fz_1max_ and Fz_2max_ (t_Fz1_, t_Fz2_), the first peak (G_1max_) and second peak (G_2max_) of LR and relative time (t_G1_, t_G2_), and LR, which is defined as the slope of the vGRF curve (the first derivative) during impact phase from initial foot contact to the maximum vGRF, see the equation below:

$$\mathrm{LR}=\underset{\Delta t}{\lim}\frac{\Delta F}{\Delta t}$$

The sagittal joint kinematic variables include the joint RoM, initial contact angle (*θ*_cont_), and minimum angle (*θ*_min_) of the hip, knee and ankle joints (Figure 4).

### 2.6. Statistics

A 2 × 2 (shoe × fatigue) two-way repeated measure ANOVA was performed to examine the existence of significant main shoe and fatigue effects and the interaction on all tested variables. When there was an interaction or a main effect, the paired *t*-tests were used as post hoc to identify potential shoes effects before or after fatigue and fatigue effects for each shoe between groups. The maximum jump height before and after fatigue-inducing intervention was analysed through one-way ANOVA (SPSS 19.0, SPSS Inc., Chicago, IL, USA.). The significance level was set to 0.05. Intraclass correlation coefficient (ICC) was used to determine the reliability of measures and was interpreted based on the thresholds: <0.49 (small), 0.50–0.69 (moderate), 0.70–0.89 (large) and 0.90–1.00 (very large) [35].

## 3. Results

### 3.1. Fatigue-Inducing Exercises

The maximum jump height was 38% lower in post-fatigue than in pre-fatigue condition. After the intervention, the participants had averaged maximum mean HR (192.4 beats/min), total fatigue-inducing exercise duration (6.2 min) and RPE (17.0), indicating that the intervention was effective in inducing an acute fatigue condition.

### 3.2. Impact Forces

**Figure 5:**
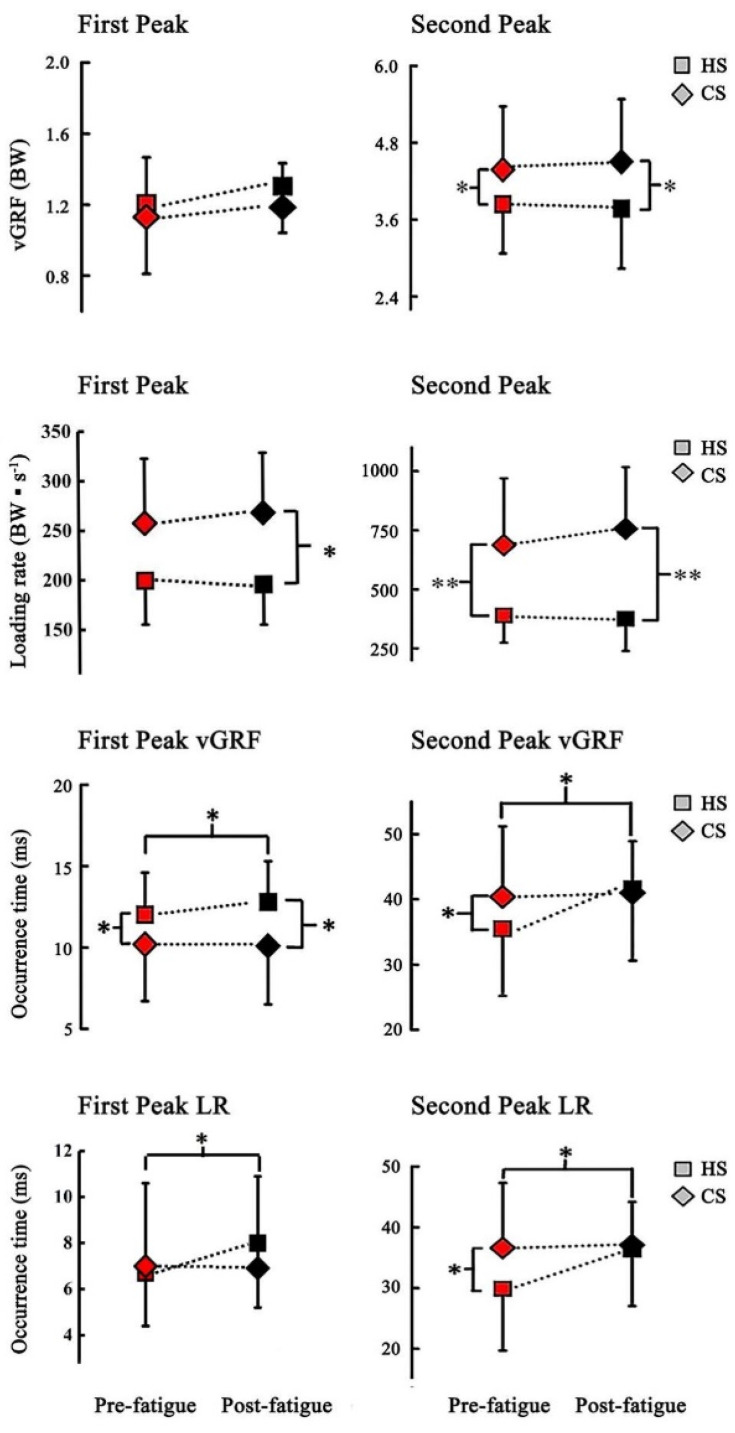
Comparison of vGRF, LR and occurrence time (t_Fz1_, t_Fz2_, t_G1_, t_G2_) between HS and less cushioned shoes (CS) in pre-fatigue and post-fatigue conditions. * indicates significant difference (*p* < 0.05). Right-pointing and left-pointing brackets indicate a significant difference between shoes in pre-fatigue and post-fatigue conditions (*p* < 0.05). ** indicates a significant difference (*p* < 0.01). Right-pointing and left-pointing brackets indicate a significant difference between shoes in pre-fatigue and post-fatigue conditions (*p* < 0.05). Upper-pointing brackets indicate a significant difference between pre-fatigue and post-fatigue conditions for HS (*p* < 0.05).

No significant interaction of shoe and fatigue was determined on Fz_1max_ (F[1,14] = 0.75, *p* > 0.05), Fz_2max_ (F[1,14] = 1.41, *p* >0.05), G_1max_ (F[1,14] = 1.03, *p* >0.05) and G_2max_ (F[1,14] = 3.88, *p* > 0.05). The main shoe effect was observed on impact force (Fz_2max_: F[1,14] = 8.50, *p* < 0.01) and LR (G_1max_: F[1,14] = 14.1, *p* < 0.05, G_2max_: F[1,14] = 37.9, *p* < 0.01) (Table 1). In particular, Fz_2max_ and G_2max_ in pre-fatigue condition was significantly lower in HS than in CS (*p* < 0.05) (Figure 5). After fatigue condition, Fz_2max_, G_1max_ and G_2max_ were significantly lower in HS than in CS (*p* < 0.05) (Figure 5). Specifically, wearing HS contributed to with 24% and 13% reduction in forefoot and rearfoot peak loading rates, respectively.

Significant interaction (F[1,14] = 6.14, *p* < 0.05) and main shoe effects were observed on t_Fz1_ (F[1,14] = 8.76, *p* < 0.01). In particular, t_Fz1_ was significantly later with HS than that of CS (*p* < 0.05), and t_Fz1_ was earlier in pre-fatigue than in post-fatigue in HS (*p* < 0.05, Table 1). Significant interaction of shoe and fatigue was observed for t_Fz2_ (F[1,14] = 10.97, *p* < 0.01), t_G1_ (F[1,14] = 10.6, *p* < 0.01) and t_G2_ (F[1,14] = 12.23, *p* < 0.01). Interaction analysis showed a significantly earlier t_Fz2_ in HS in pre-fatigue and a significantly earlier t_Fz2_ in pre-fatigue than in post-fatigue in HS (*p* < 0.05). t_G1_ was significantly earlier in pre-fatigue than in post-fatigue in HS and CS (*p* < 0.05), t_G2_ was significantly earlier in pre-fatigue than in post-fatigue in HS but no significant differences were observed between fatigue conditions in CS. t_G2_ was significantly later in CS than in HS in pre-fatigue condition (*p* < 0.05) (Figure 5).

### 3.3. Joint Kinematics

A significant interaction between shoe and fatigue was observed in knee *θ*_min_ (F[1,14] = 6.31, *p =* 0.025) and ankle *θ*_cont_ (F[1,14] = 9.07, *p* < 0.01). Main fatigue effects were observed on ankle *θ*_cont_ (F[1,14] = 7.96, *p* < 0.05). Subsequent analysis of the interaction effect indicated a lower knee *θ*_min_ in HS than in CS in pre-fatigue (*p* < 0.05) and a greater ankle *θ*_cont_ in HS in post-fatigue than in pre-fatigue condition (*p* < 0.05). However, no significant interaction was observed in *θ*_min_ of hip/ankle and *θ*_cont_ of hip/knee. Main shoe effects were observed for *θ*_cont_ of hip (F[1,14] = 8.05, *p* < 0.05) and knee *θ_cont_*_._ (F[1,14] = 6.67, *p* < 0.05). In addition, main fatigue effects were observed on ankle *θ*_min_ (F[1,14] = 4.91, *p* < 0.05). In particular, the value of *θ*_cont_ for hip and knee was significantly lower in HS than in CS in pre-fatigue (*p* < 0.05). Subsequent analysis of the interaction revealed that larger ankle *θ*_min_ was observed for cushioned shoe in post-fatigue than in pre-fatigue condition, and a smaller ankle *θ*_min_ was observed in CS than in HS under post-fatigue condition (*p* < 0.05). No significant interactions were observed on the RoM of all lower extremity joints. However, a main shoe effect was observed on RoM_hip_ (F[1,14] = 9.89, *p* < 0.01). In particular, RoM_hip_ in HS was significantly lower than in CS under post-fatigue condition (*p* < 0.05) (Table 2).

## 4. Discussion

This study examined the effects of HS on impact loading and joint kinematics during bipedal drop landing in pre-fatigue and post-fatigue conditions. During landing, participants wearing HS experienced less peak vGRF and LR of the rearfoot region regardless of fatigue states than in CS. Compared with pre-fatigue, the HS reduced the peak LR and delayed the occurrence times of peak first vGRF, first and second LRs. Greater ankle *θ*_min_ and *θ*_cont_ but a lower hip RoM were observed in HS than in CS.

### 4.1. Impact Forces

The first and second peak vGRF refer to the peak vGRF of forefoot and rearfoot contacts, respectively [20]. The current findings showed no significant differences of the forefoot peak vGRF between HS and CS in pre- and post-fatigue conditions, which is in agreement with the study of Milani, et al. [36] and Lam et al. [37]. They found that the first peak vGRF did not alter across midsole hardness. By contrast, we found a greater rearfoot peak vGRF in CS than in HS. This finding suggested that HS would play an important role in shoe attenuation at heel contact, which is consistent with the findings from previous studies [11,19]. Considering that the rearfoot peak impact was higher than the forefoot and few soft tissue structures (e.g., heel pad) attenuated the impact [38], the footwear cushioning effect was essential in reducing the impact during rearfoot contact and potentially lowering the injury risk [19]. Therefore, HS could be recommended to reduce impact forces in the rearfoot region in both pre- and post-fatigue conditions.

The LR was considered a factor of overuse injury and an important indicator for footwear cushioning [39]. The risk of overuse injuries might be associated with high LR [40]. Cushioned shoes were shown to effectively reduce LRs in the previous studies [12,31]. The findings of the present study showed that G_1max_ was significantly lower in HS than in CS under pre-fatigue condition, and G_2max_ was significantly lower in HS than in CS under pre- and post-fatigue conditions. This finding indicated that HS could provide cushioning benefits at rearfoot in both fatigue conditions. Moreover, cushioned shoes could minimise the peak plantar pressure located at first metatarsophalangeal and heel regions during basketball lay-up [41]. Compared with pre-fatigue condition, HS showed a larger reduction in LR in post-fatigue condition. Considering that the results showed a low G_1max_ when wearing HS in post-fatigue condition, the attenuation differences between HS and CS exhibited an increasing trend in post-fatigue than in pre-fatigue condition (*p* = 0.068). These results are consistent with previous studies that reported that footwear cushioning reduces peak LRs during landings from unexpected but not self-initiated drops [6]. Similar to unanticipated landing where the neuromuscular activity is detrimental, the footwear cushioning effect becomes discriminative. Hence, HS appears to be effective in preventing lower extremity injuries to compensate the diminished neuromuscular control in post-fatigue. Furthermore, the cushioning materials and structures not only plays an essential role on impact force but also the plantar pressure. Previous study reported the cushioned shoes showed overall lower plantar pressure than less cushioned shoes during landings [42]. Moreover, a significant difference was noted on the plantar pressure of two cushioned shoes during different basketball maneuvers due to the differences in the impact absorption ability of the midsole [43].

The occurrence times of peak vGRF and LR were important indicators for footwear cushioning performance. A later occurrence time indicated that attenuating the vGRF and LR can reduce impact-related injuries [44]. The results showed that t_Fz2_, t_G1_ and t_G2_ significantly increased when wearing HS under post-fatigue condition. These findings implied that t_Fz_ and t_G_ are more effectively prolonged with HS in post-fatigue than in pre-fatigue. The decreased peak LR is associated with the increased cushioning time in HS, thereby supporting that the cushioning function of shoes is discriminative when participants are under physical fatigue. Hence, wearing cushioned shoes made a great contribution to reduce the injury risk potential after acute fatigue is induced, as indicated by prolonged peak time of vGRF and LR.

Collectively, this study firstly reported the new variables (occurrence time to peak vGRF or to peak LR) to examine the shoe effects on GRF loading and found significant attenuation effect, especially on prolonged strenuous exercises in basketball. The footwear benefits can be observed after fatigue-inducing exercises on the impact forces at forefoot and rearfoot during landing.

### 4.2. Joint Kinematics

The significant shoe effects on knee and hip mechanics (e.g., *θ*_cont_) were observed in pre-fatigue condition. This finding suggests a compensatory of “soft landing” strategy in HS. The effect of RoM_hip_ was significantly affected by shoe condition when fatigue was induced, indicating that no extra effort was needed to increase RoM for attenuating and preventing the body to collapse whilst wearing HS. Compared with CS, HS increased the contact and minimum angle of ankle joint in post-fatigue without the change in RoM_ankle_, which is consistent with previous research [34]. The change in the difference of contact and minimum angle of ankle might be related to shoe structures, such as shoe collar height and shoe upper stability or tongue [45], rather than the function of the cushioned structure. The lack of non-significance between shoes requires investigation.

Only ankle angle (*θ*_cont_ and *θ*_min_) of HS was affected by fatigue by comparing the shoe performance between fatigue intervention. The knee and hip angles (*θ*_cont_ and *θ*_min_) were similar to the findings of Whyte, et al. [46]. They found no significant changes in joint angles at the initial landing phase. The significant changes in ankle angle (*θ*_cont_ and *θ*_min_) and increasing trends for hip and knee were found in HS under fatigue condition. These findings might be attributed to the cushioned shoes, which have greater heel-to-drop and would facilitate the body to flex lower limb angles after fatigue. The joint RoM related to fatigue intervention was inconsistent across previous studies [24,25,47]. Therefore, the relation of fatigue intervention to joint kinematics should be investigated before a viable conclusion can be made [30].

The highlights of this research are as follows: (1) shoe cushioning can reduce rearfoot impact forces regardless of fatigue conditions; (2) shoe interventions are important in protecting the locomotor system against impact loading during prolonged exercise; (3) compared with pre-fatigue, wearing highly-cushioned shoes contributed to a reduction in forefoot peak loading rates and later occurrence time of impacts; (4) in a situation where the neuromuscular activity is reduced or absent, e.g., post-fatigue, wearing better cushioning shoes showed a superior attenuation.

Several limitations were found in this study. Firstly, landing biomechanics was only collected in pre- and post-fatigue conditions. Different fatigue states (mild, moderate and severe) could be collected to identify the trend and threshold of significant changes. Secondly, participants might partially recover from fatigue-inducing exercises during the landing evaluation progress, thereby influencing the results in this study. Thirdly, the participants were all male basketball athletes. Considering to reducing the sampling bias, the shoes cushioning effect of impact force and lower extremity kinematics for the female basketball player or the basketball player in the different level could be further explored. In addition, plantar pressure distribution or the numerical models could also be further considered in the future. Finally, the experimental setting was performed on a standard force platform not a basketball pitch.

## 5. Conclusions

During bipedal drop landing, both rearfoot peak vGRF and LR were significantly reduced when participants wore HS in pre- and post-fatigue conditions. Compared with pre-fatigue, wearing HS contributed to the reduced forefoot peak LRs and later occurrence time of forefoot and rearfoot GRF loadings. These preliminary findings suggested that footwear cushioning can reduce landing-related rearfoot impact forces regardless of fatigue conditions. In a situation where the neuromuscular activity is reduced or absent, i.e., post-fatigue, wearing better cushioning shoes showed a superior attenuation, as indicated by low forefoot and rearfoot impacts. Such benefits of HS can minimise sports injuries, especially on a post-exercise condition.

## Figures and Tables

**Table 1:** Vertical GRF (vGRF) and loading rate (LR) of the lower extremity based on shoe conditions in pre-fatigue and post-fatigue.

	Variables	Pre-Fatigue		Post-Fatigue		ICC	CI 95%	
		HS	CS	HS	CS			
vGRF	Fz_1max_ (BW)	1.2 ± 0.3	1.1 ± 0.3	1.3 ± 0.3	1.2 ± 0.3	0.913	0.809	0.967
	t_Fz1_ (ms)	12.0 ± 2.6 *	10.2 ± 3.5	12.8 ± 2.5 *^,#^	10.1 ± 3.6	0.882	0.742	0.956
	Fz_2max_ (BW)	3.8 ± 0.8 *	4.4 ± 1.0	3.8 ± 0.9 *	4.5 ± 1.0	0.803	0.568	0.926
	t_Fz2_ (ms)	35.5 ± 10.3 *	40.4 ± 10.8	41.6 ± 7.3 ^#^	41.0 ± 10.4	0.913	0.810	0.967
LR	G_1max_ (BW·s^−1^)	199.2 ± 44.1	257.4 ± 65.0	194.5 ± 40.5 *	267.0 ± 60.2	0.693	0.308	0.89
	t_G1_ (ms)	6.7 ± 2.3	7.0 ± 3.6	8.0 ± 2.8 ^#^	6.9 ± 4.0 ^#^	0.877	0.732	0.956
	G_2max_ (BW·s^−1^)	389.7 ± 115.5 *	688.0 ± 281.4	375.5 ± 136.6 *	756.8 ± 260.0	0.766	0.482	0.912
	t_G2_ (ms)	29.9 ± 10.2 *	36.6 ± 10.7	36.4 ± 7.8 ^#^	37.1 ± 10.1	0.915	0.814	0.968

Note: Fz_1max_ represents the first peak vGRF, Fz_2max_ represents the second peak vGRF, G_1max_ represents the first peak LR, G_2max_ represents the second peak LR, and tF_z1_, tF_z2_, t_G1_, t_G2_ represent the occurrence times of the first and second peak vGRF and LR. * denotes the significance level (*p* < 0.05) between different shoe conditions under the same fatigue condition, and ^#^ denotes the significance levels (*p* < 0.05) between pre- and post-fatigue under the same shoe condition.

**Table 2:** Contact angle (*θ*_cont_), minimum angle (*θ*_min_) and RoM of hip, knee and ankle joints based on shoe conditions in pre-fatigue and post-fatigue.

Joint	Variables	Pre-Fatigue		Post-Fatigue		ICC	CI 95%	
		HS	CS	HS	CS			
Hip	*θ*_cont_ (°)	139.4 ± 5.9 *	143.4 ± 7.0	141.2 ± 7.2	143.3 ± 7.4	0.815	0.594	0.931
	*θ*_min_ (°)	86.3 ± 12.5	87.2 ± 11.2	88.8 ± 11.1	86.6 ± 11.0	0.921	0.826	0.970
	RoM_hip_ (°)	53.1± 13.2	56.2 ± 13.4	52.4 ± 12.3 *	56.7 ± 9.8	0.944	0.878	0.979
Knee	*θ*_cont_ (°)	158.6 ± 7.2 *	161.8 ± 5.6	160.3 ± 8.8	161.7 ± 5.7	0.888	0.755	0.958
	*θ*_min_ (°)	72.0 ± 21.0 *	77.7 ± 17.0	74.7 ± 22.6	76.1 ± 18.6	0.979	0.954	0.992
	RoM_knee_ (°)	86.6 ± 18.0	84.1 ± 16.9	85.4 ± 23.1	85.6 ± 17.1	0.944	0.881	0.978
Ankle	*θ*_cont_ (°)	126.5 ± 10.0 ^#^	128.1 ± 8.1	131.7 ± 8.4	129.8 ± 6.9	0.923	0.831	0.971
	*θ*_min_ (°)	84.7 ± 7.2 ^#^	84.5 ± 5.3	87.0 ± 7.8	85.2 ± 6.9	0.971	0.937	0.989
	RoM_ankle_ (°)	41.8 ± 9.8	43.6 ± 8.3	43.3 ± 11.9	44.6 ± 7.7	0.923	0.832	0.971

Note: *θ*_cont_ represents the joint contact angle, *θ*_min_ represents the minimum angle, and RoM represents the joint RoM. * indicates the significance level (*p* < 0.05) between shoe conditions under the same fatigue condition, and ^#^ denotes the significance level (*p* < 0.05) in pre-fatigue and post-fatigue under the same shoe condition.

## Data Availability

The data presented in this study are available on request from the corresponding author. The data are not publicly available due to restrictions e.g., privacy or ethical.
